# Pan-cancer analysis identifies LPCATs family as a prognostic biomarker and validation of LPCAT4/WNT/β-catenin/c-JUN/ACSL3 in hepatocellular carcinoma

**DOI:** 10.18632/aging.204723

**Published:** 2023-05-23

**Authors:** Yaoyong Lu, Hongfeng Liang, Xiaoyin Li, Haiwen Chen, Changfu Yang

**Affiliations:** 1 Department of Oncology (Section 3), Gaozhou People’s Hospital, Gaozhou, Guangdong, China; 2 The Second Affiliated Hospital of Guangzhou University of Chinese Medicine (Guangdong Provincial Hospital of Chinese Medicine) Guangzhou, Guangdong, China

**Keywords:** LPCATs, bioinformatics, liver hepatocellular carcinoma, cholesterol biosynthesis

## Abstract

Lipid remodeling regulators are now being investigated as potential therapeutic targets for cancer therapy as a result of their involvement, which includes promoting cancer cells’ adaptation to the restricted environment. Lysophosphatidylcholine acyltransferases (LPCATs, LPCAT1-4) are enzymes that regulate the remodeling of bio-membranes. The functions of these enzymes in cancer are largely unknown. In the current study, we found that genes belonging to the LPCAT family participated in tumor advancement and were strongly linked to dismal prognosis in many different malignancies. We constructed the LPCATs scores model and explored this model in pan-cancer. Malignant pathways in pan-cancer were positively related to LPCATs scores, and all pathways had strong links to the tumor microenvironment (TME). Multiple immune-associated features of the TME in pan-cancer were likewise associated with higher LPCATs scores. In addition, the LPCATs score functioned as a prognostic marker for immune checkpoint inhibitor (ICI) therapies in patients with cancer. LPCAT4 enhanced cell growth and cholesterol biosynthesis by up-regulating ACSL3 in hepatocellular carcinoma (HCC). WNT/β-catenin/c-JUN signaling pathway mediated LPCAT4’s regulation on ACSL3. These findings demonstrated that genes in the LPCAT family might be used as cancer immunotherapy and prognosis-related biomarkers. Specifically, LPCAT4 could be a treatment target of HCC.

## INTRODUCTION

Lysophosphatidylcholine acyltransferases (LPCATs) are a family of enzymes consisting of four isoforms (LPCAT1-4) in humans that catalyze the conversion of lysophosphatidylcholine (LPC) to phosphatidylcholine (PC) [1]. The PCs are the most prevailing kind of lipids found in cell membranes [2]. The ability of cancerous cells to adapt to the local tumor microenvironment (TME) is facilitated by PC metabolic dysregulation and the resulting modifications in membrane composition, which may supply energy, epigenetic regulators, and signaling molecule substrates [3]. Considering the crucial role played by LPCATs in PC formation, reports have documented that LPCATs are involved in the emergence of cancers [4]. For instance, overexpression of LPCAT1 was implicated in clear cell renal cell carcinomas, prostate cancer, esophageal squamous cell carcinoma and lung cancer [5]. LPCAT2 and LPCAT4 contributes to initiation and chemoresistance in colorectal cancer [6]. LPCAT3 participates in acute myeloid leukemia progression [7].

In this research, we systematically analyzed the LPCAT family of genes across 32 different tumor types for alterations (single nucleotide variation, copy number variation), expression, clinical characteristics, and prognostic significance. We also specifically explored LPCAT4’s biological function in LIHC and uncovered the underlying mechanism.

## RESULTS

### LPCATs family genes expression distribution in pan-cancer

**Figure 1:**
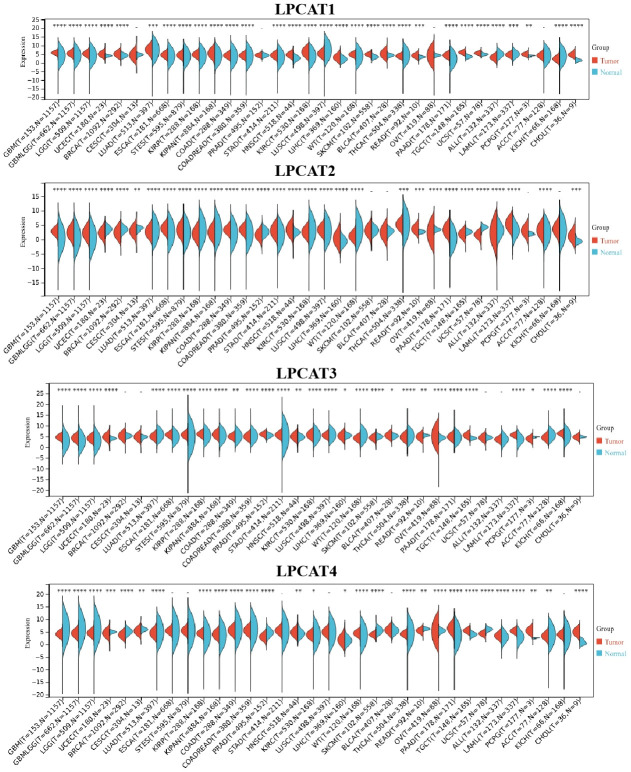
LPCATs family genes expression distribution in pan-cancer.

We analyzed LPCATs family genes (LPCAT1-4) expression levels in pan-cancer. It was found that LPCAT1-4 expression levels were elevated in most kinds of cancer types (Figure 1).

### Gene alterations of LPCATs family genes

**Figure 2:**
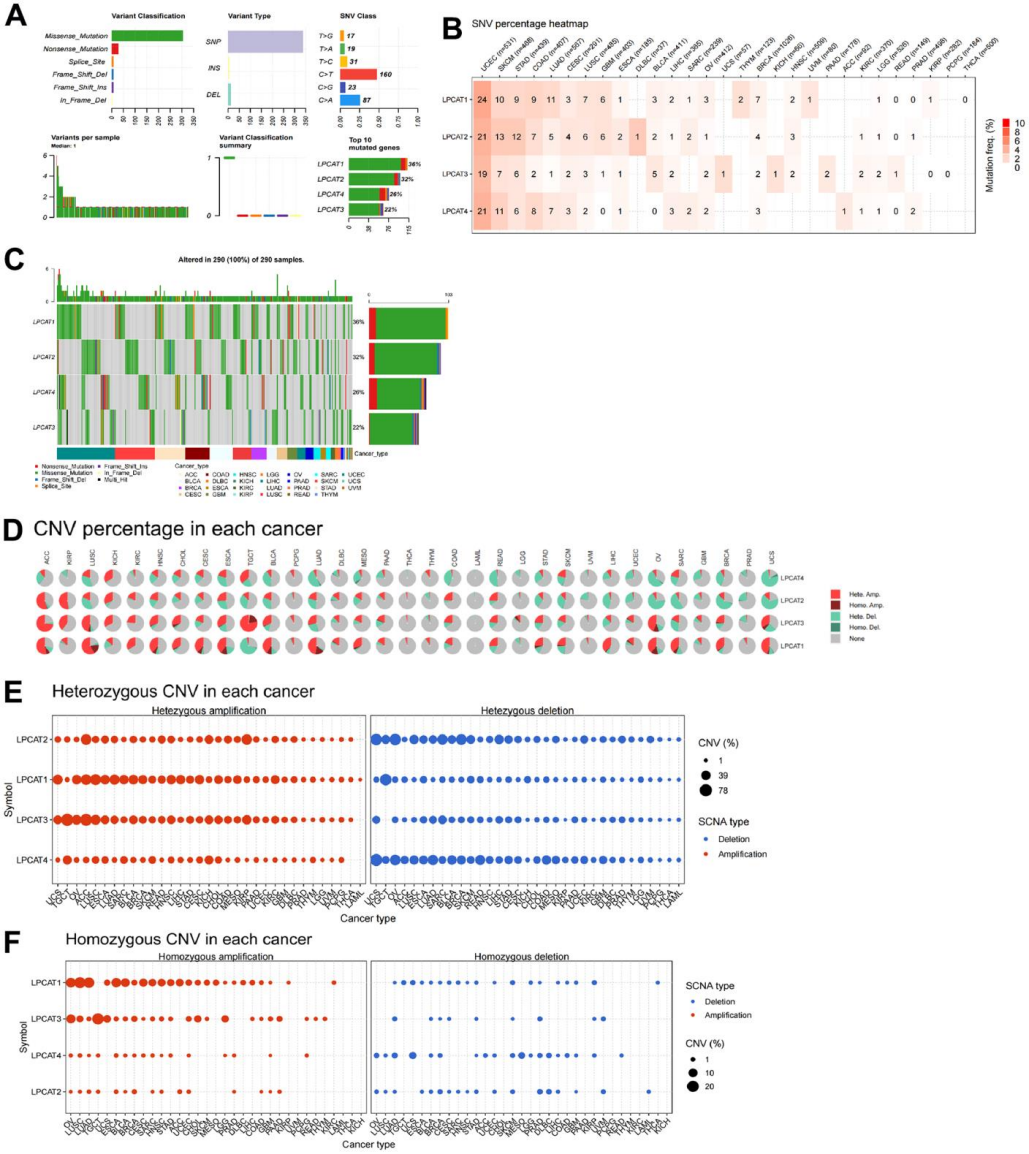
**Single-nucleotide variants and gene copy number variants (CNV) of LPCATs family genes in pan-cancer.** (**A**) Overall perspective of LPCAT1-4 genes was demonstrated in pan-cancer. (**B**) The single-nucleotide variant status of LPCAT1-4 genes in pan-cancer was showed. (**C**) The mutation frequency of LPCAT1-4 genes in pan-cancer was demonstrated. (**D**) The proportion of different types of CNV of LPCATs family genes in pan-cancer was demonstrated. (**E**) The CNV of LPCAT family genes heterozygous amplification and deletion were demonstrated. (**F**) The CNV of LPCAT family genes homozygous amplification and deletion were demonstrated.

We studied the genetic alterations that occurred in the LPCAT1-4 genes and how those alterations were linked to mRNA levels. We discussed the top10 mutant genes, variant classification, single-nucleotide variant (SNV) class, variant type, variants per sample, and variant classification (Figure 2A). For the SNV percentage, uterine corpus endometrial carcinoma (UCEC) had the greatest frequency of mutations in the LPCAT family of genes, as per the total deleterious mutation percentage (Figure 2B). In addition, we observed that among the LPCAT family genes, LPCAT1 exhibited the greatest mutation frequency (36%) (Figure 2C).

Additional research on LPCAT family gene copy number variants (CNV) was conducted. The proportion of different types of CNV (including heterozygous amplification, heterozygous deletion, homozygous amplification, and homozygous deletion) of each gene in pan-cancer was shown (Figure 2D). The CNV of LPCAT family genes heterozygous amplification and deletion were demonstrated in Figure 2E. In addition, The CNV of LPCAT family genes homozygous amplification and deletion were demonstrated in Figure 2F.

### Construction of LPCATs score and its value in pan-cancer

**Figure 3:**
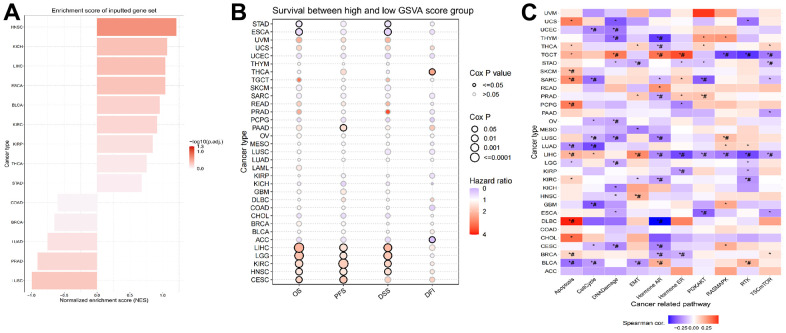
**Construction of LPCATs score and its value in pan-cancer.** (**A**) The ssGSEA analysis was used to construct LPCATs score in pan-cancer. (**B**) The uniCox analysis was used to evaluate association among OS, PFS, DSS, DFI and LPCATs score. (**C**) The correlation between the LPCATs score and the GSVA score in cases of pan-cancer.

To approximate the LPCATs score in the TCGA cohort, we used ssGSEA. HNSC had the greatest and LUSC had the least LPCATs score (Figure 3A). Also, the LPCATs score was elevated in tumors compared with normal tissues for BLCA, ESCA, HNSC, KICH, KIRC, KIRP, LIHC, STAD, and THCA, but lower for BRCA, LUAD, LUSC, and PRAD (Supplementary Figure 1A).

We further analyzed the association between LPCATs score and survival rate. The findings of the uniCox analysis are as follows: (1) In terms of OS, LPCATs score served as a risk factor in LIHC, LGG, KIRC, HNSC, and CESC, and a protective factor in ESCA and STAD; (2) For PFS, LPCATs score was a risk factor in LIHC, CESC, KIRC, HNSC, LGG, and PAAD; (3) In terms of DSS, LPCATs score functioned as a risk factor in LIHC, LGG, KIRC, and HNSC, but a protective factor in ESCA and STAD; (4) In terms of DFI, LPCATs score functioned as a risk factor in THCA, whereas a protective factor in ACC (Figure 3B and Supplementary Table 1).

### GSVA analysis of LPCATs score in pan-cancer

We carried out a GSVA premised on HALLMARK pathways to determine the prospective pathways that were influenced by the LPCATs score. Figure 3C illustrates the correlation between the LPCATs score and the GSVA score in cases of pan-cancer. Some key signaling pathways were found significantly associated with LPCATs score. For instance, apoptosis and cell cycle were important for the cell growth of cancer. Epithelial to mesenchymal transition (EMT) contributed to cancer cell migration and invasion. Hormone AR and ER were tightly related with endocrine therapy. PIK3/AKT signaling pathway ranked as one of the most common abnormal pathways in cancer. LPCATs score could be a useful method in predicating the abnormal pathways across pan-cancer.

### Analysis of association between LPCATs score and immune infiltrating

**Figure 4:**
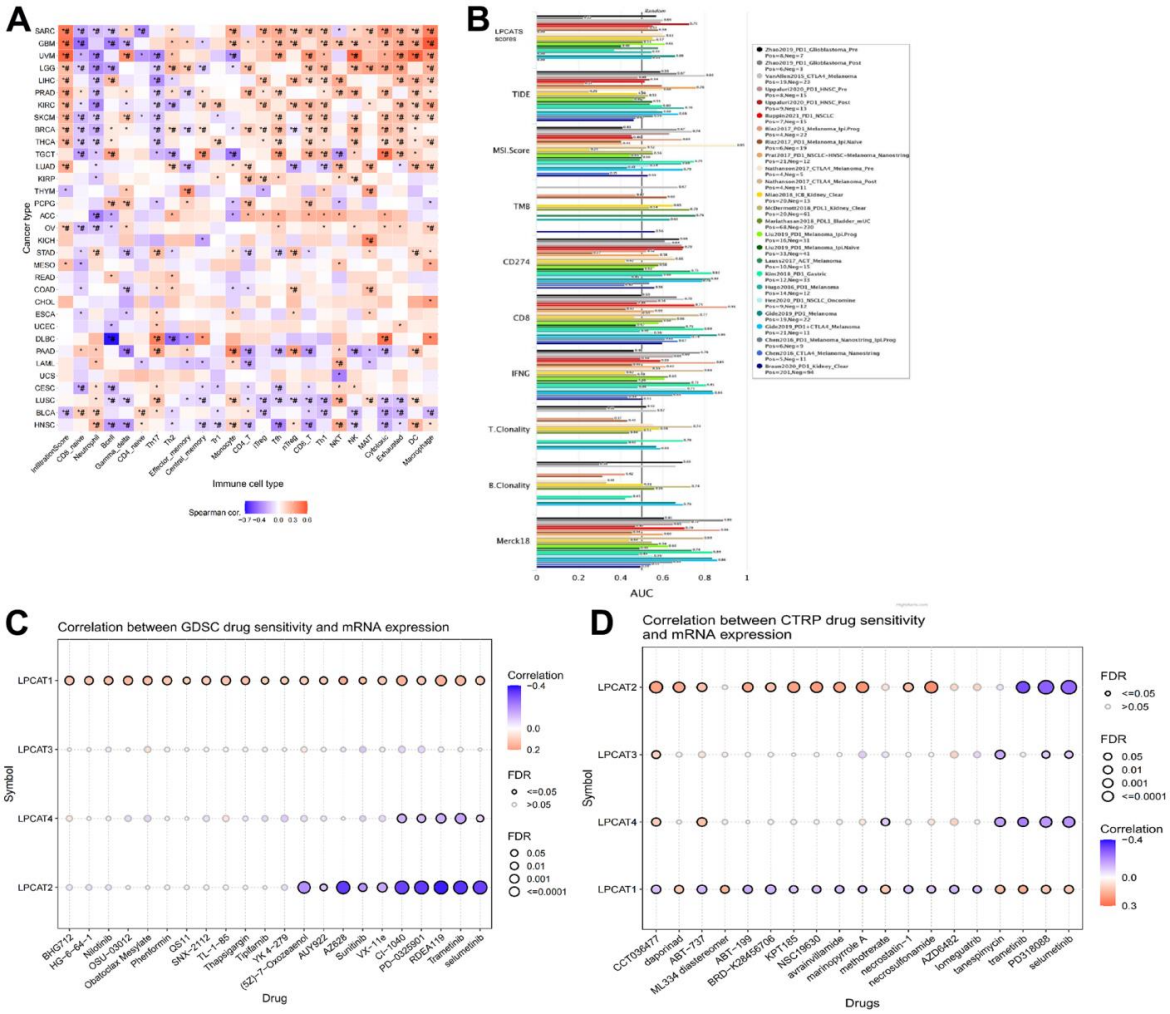
**Analysis of association between LPCATs Score and immune infiltrating, potent anticancer inhibitors.** (**A**) The link between LPCATs Score and immune infiltrating score in pan-cancer was illustrated. (**B**) The value of LPCATs Score in predicating response outcomes and OS in immune checkpoint blockade (ICB) sub-cohorts was demonstrated. (**C**) The link between gene expression and the sensitivity of GDSC pharmaceuticals (top 30) was represented. (**D**) The top 30 CTRP pharmaceuticals and their sensitivity to pan-cancer expression profiles are presented.

We evaluated the link between LPCATs Score and immune infiltrating score in pan-cancer. It was found that a high LPCATs Score predicated a high level of immune infiltrating in SARC, GBM, and UVM, which ranked top three in pan-cancer. Additionally, the LPCATs score had a strong link to the majority of immune cells, suggesting that the TME was immune-activated (Figure 4A).

We examined the significance of the LPCATs Score as a biomarker by making a comparison of it with standard biological markers premised on their ability to accurately predict response outcomes and OS in immune checkpoint blockade (ICB) subcohorts. We determined that the LPCAT Score alone had an area under the receiver operating characteristic curve (AUC) of more than 0.5 in 17 of the 20 ICB subcohorts (Figure 4B). The LPCATs Score had a greater prognostic significance as opposed to TMB, T.Clonality, and B.Clonality, each of which had AUC values of more than 0.5 in eight, nine, and seven ICB subcohorts, respectively. However, the AUC value in LPCATs was decreased in contrast with that in CD8, CD274, MSI score, and TIDE.

### Prediction of potent anticancer inhibitors of the LPCATs family genes signaling axis

We searched for powerful anticancer medications by searching databases called GDSC (Genomics of Drug Sensitivity in Cancer) and CTRP (The Cancer Therapeutics Response Portal). These databases included information on pharmaceuticals whose unique mechanism of action involves the suppression of LPCATs family genes. In pan-cancer, the link between gene expression and the sensitivity of GDSC pharmaceuticals (top 30) was represented in Figure 4C. The top 30 CTRP pharmaceuticals and their sensitivity to pan-cancer expression profiles are presented in Figure 4D. Some of the predicated drugs (e.g. trametinib, sunitinib, selumetinib, nilotinib) are already widely used in the treatment of cancers.

### Identification of LPCAT4 biological function in LIHC

**Figure 5:**
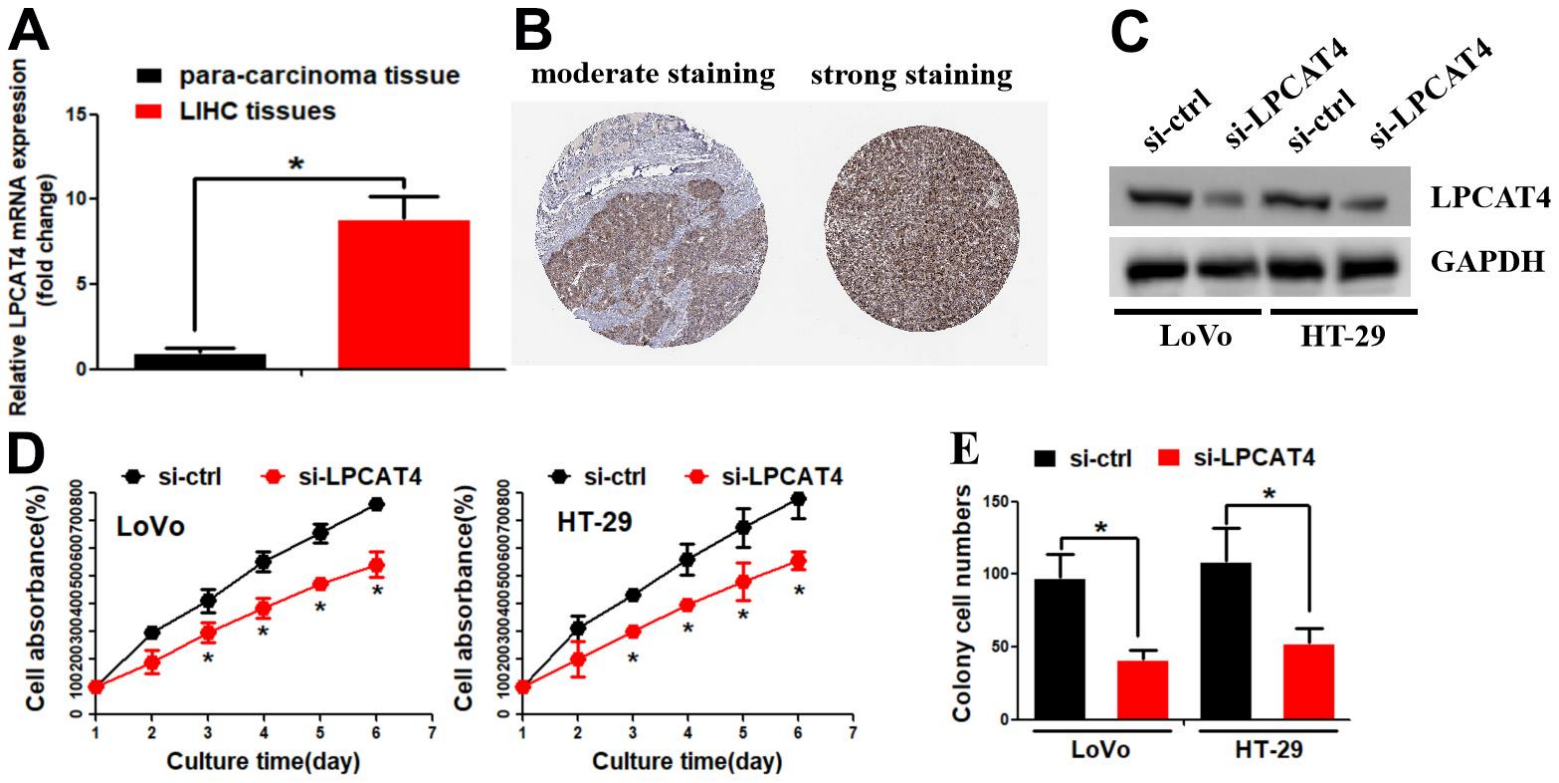
**Identification of LPCAT4 biological function in LIHC.** (**A**) LPCAT4 mRNA expression level was examined by RT-PCR assay. (**B**) LPCAT4 protein expression level in LIHC tissues. (**C**) Western blot assay was used to examine LPCAT4 protein down-regulation efficiency. (**D**) MTT assay was used to analyze cell growth. (**E**) Colony formation ability in LPCAT4 down-regulation and control group.

The above findings revealed that LPCATs score was a risk factor in the OS of LIHC, we thus further explored the LPCATs family members’ function in LIHC. Our PCR assay revealed that LPCAT4 mRNA expression level was significantly elevated in LIHC samples (Figure 5A). In parallel, the Human Protein Atlas (HPA) database revealed that LPCAT4 protein expression level was elevated in LIHC tissues (Figure 5B). With the use of the Cancer Cell Line Encyclopedia (CCLE) database, we uncovered that LPCAT4 was expressed in a majority of LIHC cell lines (Supplementary Figure 1B). We employed previously developed LIHC cells that stably expressed low levels of LPCAT4 to investigate its biological role in LIHC (Figure 5C). Cell proliferation was evaluated by means of MTT and colony formation assays. As expected, down-regulation of LPCAT4 decreased cell growth and colony formation ability, when compared with negative control (Figure 5D, 5E).

### Functional enrichment analysis of LPCAT4 in LIHC

**Figure 6:**
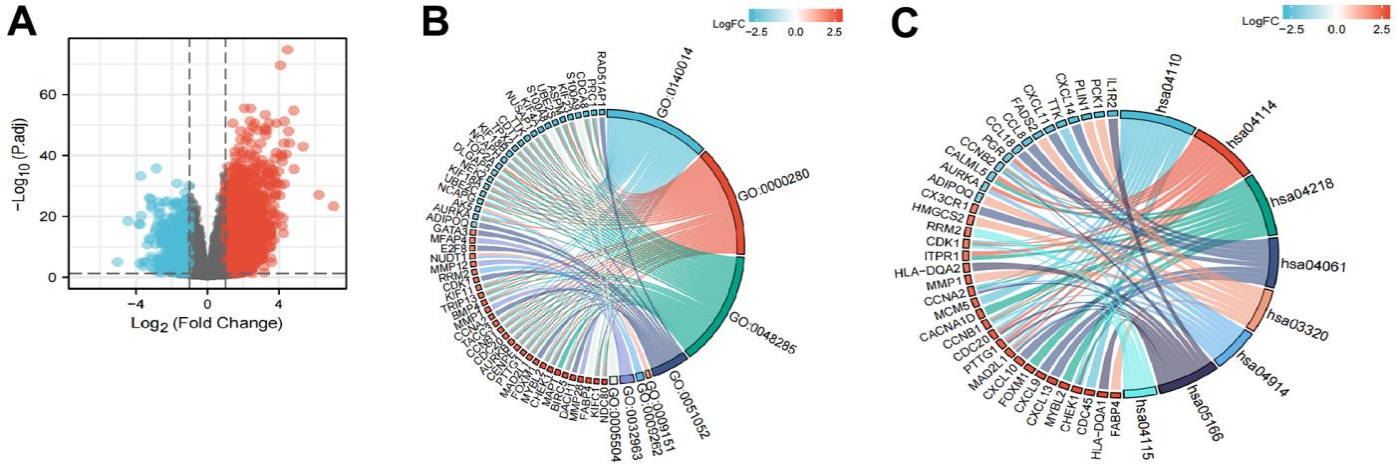
**Functional enrichment analysis of LPCAT4 in LIHC.** (**A**) Volcano plot indicated the significantly down-regulated and up-regulated DEGs. (**B**) GO analysis of DEGs. (**C**) KEGG analysis of DEGs.

A total of 2731 genes were differentially expressed between the groups with high and low expression levels of LPCAT4 (adjusted p value <0.05, |Log2-FC| > 1, Figure 6A). We then analyzed how these DEGs were enriched in GO enrichment analysis (including biological processes, cellular compositions, and molecular functions). The results showed that DEGs were enriched in different GO terms such as mitotic nuclear division, nuclear division, regulation of DNA metabolic process, purine deoxyribonucleotide metabolic process, deoxyribonucleotide metabolic process and collagen metabolic process (Supplementary Table 2 and Figure 6B). In addition, KEGG pathway analysis showed that significantly DEGs-enriched pathways included cell cycle, oocyte meiosis, cellular senescence and PPAR signaling pathway (Supplementary Table 2 and Figure 6C).

### LPCAT4 increased the expression levels of cholesterol biosynthesis via up-regulating ACSL3

**Figure 7:**
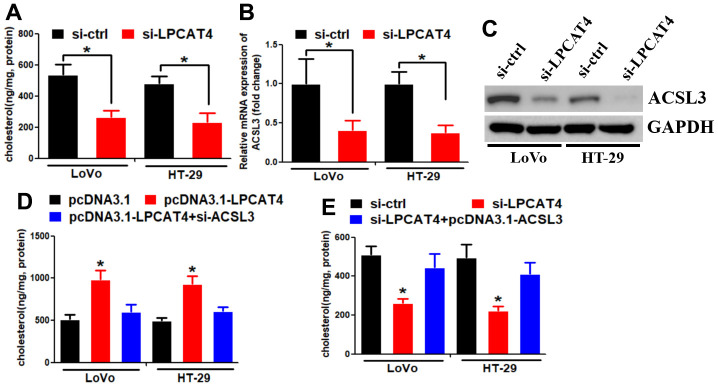
**LPCAT4 increased the expression levels of cholesterol biosynthesis via up-regulating ACSL3.** (**A**) Cholesterol synthesis ability was analyzed in LPCAT4 down-regulation and control group. (**B**, **C**) ACSL3 mRNA and protein expression levels were analyzed by RT-PCR and western blot assays, respectively. (**D**) Overexpression of LPCAT4 increased cholesterol biosynthesis, while knockdown of ACSL3 dismissed this effect. (**E**) Down-regulation of LPCAT4 decreased cholesterol biosynthesis, while overexpression of ACSL3 could counteract this effect.

We assessed the levels of cholesterol in LIHC cells and observed a reduction in cholesterol synthesis when LPCAT4 was down-regulated (Figure 7A). A mass spectrometric analysis published in Biogrid (https://thebiogrid.org) [8] showed that ACSL3 interacted with LPCAT4. Because ACSL3 was a cholesterol biosynthesis enzyme, we thus asked whether ACSL3 mediated LPCAT4’s function on cholesterol biosynthesis. Indeed, we observed that LPCAT4 down-regulation inhibited ACSL3 mRNA and protein expression levels (Figure 7B, 7C). Overexpression of LPCAT4 increased cholesterol biosynthesis, while knockdown of ACSL3 dismissed this effect (Figure 7D). On the contrary, down-regulation of LPCAT4 decreased cholesterol biosynthesis, while overexpression of ACSL3 could counteract this effect (Figure 7E). These data implicated that ACSL3 was involved in mediating LPCAT4’s function on cholesterol metabolism.

### LPCAT4 regulated ACSL3 expression via WNT/β-catenin/c-JUN signaling pathway

**Figure 8:**
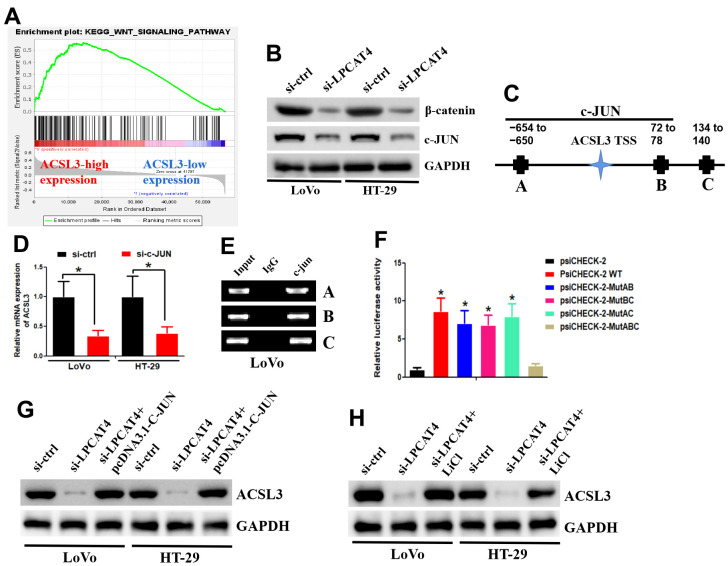
**LPCAT4 regulated ACSL3 expression via WNT/β-catenin/c-JUN signaling pathway.** (**A**) GSEA analysis indicated that WNT signaling pathway was found to be significantly enriched in the high LPCAT4 expression group. (**B**) Western blot assay was used to examine protein expression level. (**C**) The three transcription factor-binding sites of c-JUN on potential ACSL3 promoter region were indicated. (**D**) RT-PCR was used to examine ACSL3 mRNA expression. (**E**) The ChIP assay was used to validate binding domains of c-JUN in the potential ACSL3 promoter. (**F**) The luciferase assay was used to confirm which binding sites were functional. (**G**, **H**) Western blot assay was used to examine protein expression level.

GSEA was applied between the high- and low-LPCAT4 expression groups, and WNT signaling pathway was found to be significantly enriched in the high LPCAT4 expression group (Figure 8A). The western blot assay revealed that down-regulation of LPCAT4 inhibited WNT/β-catenin signaling activity and its downstream target c-JUN (Figure 8B). The UCSC and PROMO bioinformatics software packages were utilized to analyze a region that was located -800 bp upstream of the transcriptional start site of ACSL3. This was done to assess the transcription regulatory mechanisms that regulate the expression of ACSL3. Within the potential ACSL3 promoter region, three c-JUN-binding motifs were detected at positions -654 to -660, 72 to 78, and 134 to 140, respectively. The names A, B, and C were given to these three transcription factor-binding sites (TFBSs) (Figure 8C). To probe into the role that c-JUN performs in the regulation of ACSL3, we began by inhibiting c-JUN expression in LIHC cells with the help of small interfering RNAs (siRNAs). Next, qPCR testing revealed that the level of ACSL3 expression in LIHC cells had been remarkably reduced as a result of c-JUN knockdown (Figure 8D), illustrating that c-JUN serves as an upstream regulator of ACSL3. In LIHC cells, the c-JUN protein was shown to be recruited to all three binding domains in the potential ACSL3 promoter as per ChIP assays (Figure 8E). In addition, the luciferase assay was performed to further confirm which binding sites were functional. As illustrated in Figure 8F, by co-transfected with c-JUN plasmid, luciferase activity in the wild-type ACSL3 promoter was enhanced in LIHC cells. A similar effect was observed in ACSL3 promoter with different mutant sites (mutating sites A and B, sites A and C, and sites B and C) co-transfected with c-JUN plasmid (Figure 8F). However, a reduction of luciferase activity was observed in ACSL3 promoter with mutant sites (mutating sites A, B and C) co-transfected with c-JUN plasmid (Figure 8F). Collectively, these findings illustrate that c-JUN participates in ACSL3 transcription, and the three c-JUN binding domains in the ACSL3 promoter are all active in LIHC cells.

In addition, down-regulation of LPCAT4 could decrease ACSL3 expression level, while overexpression of c-JUN dismissed this effect (Figure 8G). Similarly, down-regulation of LPCAT4 could decrease ACSL3 expression level, while addition of LiCl (Wnt/β-catenin pathway activator) counteracted this effect (Figure 8H).

Taken together, these data implicated that the Wnt-β-catenin-c-JUN pathway may be involved in mediating LPCAT4 regulation on ACSL3 expression.

## DISCUSSION

The lysophosphatidylcholine acyltransferases (LPCATs) family contains four members, namely LPCAT1-4 [9]. LPCATs are crucial in lipid metabolism since they allow for the production of novel phospholipid molecules in a way that is separate from de novo synthesis [10]. Recently, it has been reported that LPCATs family participated in cancer progression [11]. For instance, prostate cancer that is resistant to castration advances due to LPCAT1’s ability to enhance mRNA synthesis as well as PAF production [12]. The production of lipid droplets via the LPCAT2 pathway promoted chemoresistance in colorectal cancer [13]. LPCAT3 exerted a role in both the survival of macrophages and pro-tumorigenic activity [14]. These literates prompted us to investigate LPCATs family genes’ function in pan-cancer.

With the aid of the TCGA and GTEx datasets, we evaluated the differential expression and prognostic significance of genes belonging to the LPCATs family across 32 distinct kinds of tumors. We discovered that genes belonging to the LPCAT family were remarkably engaged in the growth of tumors and acted as risk factors for the majority of cancers. To get a deeper understanding of the possible carcinogenic mechanisms of genes belonging to the LPCATs family, we provided a detailed description of many gene alterations (including methylations, homozygous deletions, amplifications, fusions, and mutations) in LPCATs family genes. The results showed that these genetic alterations together contributed to the abnormal expression of LPCATs family genes.

In addition, we constructed a LPCATs score using the ssGSEA approach in pan-cancer. The levels of LPCATs were shown to be elevated in tumor tissue compared to surrounding normal tissue in the cases of BLCA, ESCA, HNSC, KICH, KIRC, KIRP, LIHC, STAD, and THCA, whereas they were found to be lowered in the cases of BRCA, LUAD, LUSC, and PRAD. Additionally, a high score on the LPCAT might indicate a grim prognosis for patients with many different malignancies. After conducting extensive research, we concluded that the LPCATs scores had a favorable correlation with several cancerous pathways in pan-cancer, including PI3K/AKT, epithelial-mesenchymal transition, apoptosis, and the cell cycle.

The structure and function of the tumor microenvironment (TME) are intricately linked to the advancement and metastasis of tumors [15]. In particular, a TME with immune effector cells that limit tumor progression may be utilized to evaluate the response of tumor cells to immunotherapy [16]. This method has also been successfully implemented to effectively affect clinical outcomes [17]. As a result, we additionally evaluated whether or not there was an association between the LPCATs score and immune cells in the TME. At the pan-cancer level, we discovered that the genes of the LPCAT family had a strong correlation with the majority of immune cells, which pointed to the presence of an immune-activated TME. LPCAT family participates in the reacylation step of the catalytic phospholipid remodeling process, which is highly associated with TME [18]. Previous studies demonstrated that LPCAT family members were involved in infiltration of immune cells, thereby affecting TME. For instance, LPCAT3 was correlated with immune infiltration in acute myeloid leukemia [7]. LPCAT1 acts as an independent prognostic biomarker correlated with immune infiltration in hepatocellular carcinoma [19]. Our study further constructed LPCATs score and predicated that high LPCATs score was associated with immune-activated TME. We proposed that LPCATs affecting TME by modulating lysophosphatidylcholine metabolism.

In addition, our analysis indicated that LPCATs score was a good indicator for patients who received ICB treatment. High tumor mutation burden (TMB-H) has been proposed as a predictive biomarker for response to immune checkpoint blockade, largely due to the potential for tumor mutations to generate immunogenic neoantigens [20]. T.Clonality and B.Clonality predicated T or B cell immunoreactivity and sensitivity to immune checkpoint blockade [21]. Our findings revealed that LPCATs score was superior to TMB, T.Clonality and B.Clonality in predicating sensitivity to immune checkpoint blockade. All of the aforementioned findings pointed to the fact that individuals with elevated LPCATs scores had a significant amount of immune cell infiltration, which could have a beneficial effect on immunotherapy.

Taken together, the construction of LPCATs scores has important predictive significance for cancer research and prediagnosis of cancer. High LPCATs scores indicate a grim prognosis for patients with many different malignancies and have a favorable correlation with several cancerous pathways. LPCATs scores can also predicate sensitivity to pharmaceuticals and ICB treatment. Thus, with the use of LPCATs scores, we can evaluate the risk of cancer patients and treatment efficiency.

To further illustrate the biological function of LPCATs family genes, we focused on LPCAT4’s function in LIHC. Abnormal expression of LPCAT4 was involved in the progression of colorectal cancer and prostate cancer. LPCAT4 participated in transforming growth factor-β mediated alteration in phospholipid metabolism in HepG2 cells [22]. However, the biological roles of LPCAT4 in LIHC, as well as the mechanism that underlies it, were still only partially understood. Our analysis revealed that down-regulation of LPCAT4 decreased LIHC cell growth and cholesterol biosynthesis. Cholesterol is essential for membrane biogenesis and cell proliferation. Targeting cholesterol biosynthesis may be a useful way to inhibit cancer cell growth [23]. Our data revealed that inhibition of LPCAT4 decreased HCC cell growth via cholesterol biosynthesis. Mechanistically, LPCAT4 increased the expression levels of cholesterol biosynthesis via up-regulating ACSL3. ACSL3 can activate fatty acids through ATP-dependent Coenzyme A thioesterification to generate fatty acyl-CoAs. Elevated expression level of ACSL3 has been observed in HCC [24]. Activated Wnt/β-catenin signaling pathway often contributed to aberrant cholesterol metabolism [25]. We speculated that LPCAT4 modulated cholesterol biosynthesis through ACSL3. WNT/β-catenin/c-JUN signaling pathway mediated LPCAT4’s regulation of ACSL3 in LIHC.

## CONCLUSIONS

In summary, our research showed that genes belonging to the LPCAT family are risk factors for developing tumors and perform key roles in the onset and advancement of cancers. The higher LPCATs scores were shown to have a strong association with a variety of immune-related characteristics of TIME in pan-cancer. Moreover, the LPCATs score was a probable biological marker for treatment effectiveness in patients with tumors undergoing ICI therapy. In addition, we identified LPCAT4 as a new treatment target for LIHC.

## MATERIALS AND METHODS

### Overview of the analytical tools

The SangerBox (http://vip.sangerbox.com/) is a web-based platform for analyzing gene expression in TCGA. GSCALite (http://bioinfo.life.hust.edu.cn/web/GSCALite/) is a web-based program that integrated immunogenomic and genomic data of 33 different tumor types from TCGA, as well as pharmacological responsiveness from the Genomics of Drug Sensitivity in Cancer (GDSC). In addition, GSCALite was used for analyzing the correlation between LPCATs score and overall survival (OS) rate, pathway activity, and immune infiltration. TIDE (Tumor Immune Dysfunction and Exclusion, http://tide.dfci.harvard.edu/) is a web-based database used for analyzing OS rates of patients who received immune checkpoint blockade.

### Cell culture

The Cell Bank of the Chinese Academy of Sciences supplied the human LIHC cell lines employed in this study. The cell lines were grown in RPMI 1640 medium supplemented with 10% fetal bovine serum (FBS) (Biowest, Loire Valley, France). The mycoplasma-free status of all cell lines was verified, after which they were cultured at 37° C in a humidified air environment containing 5% CO2.

### Cell counting kit-8 (CCK8) assays

LIHC cells were transiently transfected at a seeding density of 2000 cells/well in 96-well flat-bottomed plates with 100 μl of growth medium. After adding 10 μl of CCK-8 solution to each well, the plates were then placed in an incubator at 37° C for 2-, 24, 48, and 72-hours, respectively. An automated microplate reader (Tecan, Switzerland) was employed to determine each well’s absorbance at 450 nm, and the results were recorded.

### Colony formation assay

To conduct the clonogenic assay, 500 cells were plated in a 6-well plate and left to grow for 14 days. In addition, 0.1% of crystal violet was used to stain the cell colonies after they had been fixed with paraformaldehyde at a concentration of 4 %. Thereafter, following the imaging process, the stained colonies were counted manually.

### Cell transfection

The design and synthesis of siRNAs for LPCAT4 and c-JUN were done by RiboBio Inc. (Guangzhou, China). LPCAT4 and c-JUN plasmids were obtained from Biosense Technologies (Guangzhou, China). PI3K inhibitor LY294002 was procured from Sigma. The transfection was carried out following the specifications provided by the manufacturer of the Reagent (Fermentas, Vilnius, Lithuania). After 48–72 hours, the cells were harvested for use in subsequent studies.

### RNA isolation and RT-PCR

We utilized the TRIzol reagent kit (Takara, Dalian, China) to isolate total RNA from the cell or tissue cultures before reverse-transcribing it into complementary DNA (cDNA) with the aid of reverse transcription reagents (TaKaRa, Shiga, Japan) following the procedures outlined by the manufacturer. For the reverse transcription polymerase chain reaction (RT-PCR) procedure, the Bio-Rad T100 equipment was used. Quantitative Real-Time PCR (qRT-PCR) was done with the SYBR Premix ExTaq (TaKaRa, Shiga, Japan). The complementary DNA was employed as a template in an amplification process using specified primers (Supplementary Table 3).

### Cholesterol synthesis test

Cholesterol was extracted from cells and fragments using a Cholesterol Assay Kit (Abcam) according to the manufacturer’s instruction, and the cholesterol content was normalized by the corresponding protein content.

### Luciferase reporter assay

A PCR-amplified 1300 base pair (bp) segment comprising the three c-JUN binding domains was introduced into a psiCHECK-2 luciferase reporter vector to construct an ACSL3 promoter vector. As a further step, vectors with mutated c-JUN binding domains were developed. Lipofectamine 2,000 Reagent (Invitrogen) was used for the purpose ofco-transfecting a psiCHECK-2 derived vector with a c-JUN expressing vector into LIHC cells.

### Chromatin immunoprecipitation (ChIP) assay

ChIP assay was done to determine whether or not c-JUN combined with the ACSL3 promoter using a CHIP assay kit as per the directions provided by the manufacturer (Millipore, catalog: 17-371). Initially, 1% formaldehyde was used to fix LIHC cells with or without ectopic c-JUN expression so that proteins could be covalently cross-linked to DNA. Next, chromatin was extracted from the LIHC cells. Sonication was done to reduce the length of the cross-linked DNA to between 200 and 1,000 bp, and it was subsequently subjected to an immunoselection procedure, which involved the use of an antibody against c-JUN (Cat. No.9165, 1:50, CST). Premised on the selected primers, PCR was subsequently utilized to determine the enrichment of DNA fragments in the potential c-JUN-binding domains located in the ACSL3 promoter (Supplementary Table 3).

### Western blotting and antibodies

SDS-PAGE (Sodium Dodecyl Sulphate - PolyAcrylamide Gel Electrophoresis) analysis was conducted on cell lysates and tumor tissues, each containing an equivalent amount of protein before being transferred to a Polyvinylidene fluoride (PVDF) membrane (Merck Life Science S.r.l., Milano, Italy). The concentration of antibodies used in the study was: LPCAT4(1:500, Abcam, Lot. ab199181), ACSL3(1:500, Abcam, Lot. ab151959), β-catenin (1:500, Abcam, Lot. ab305261), c-jun (1:500, Abcam, Lot. ab40766) and GAPDH (1:500, Abcam, Lot. ab9485).

### Data availability statement

All the data generated for this study are available on request to the corresponding author.

## Supplementary Material

Supplementary Figure 1

Supplementary Table 1

Supplementary Table 2

Supplementary Table 3

## References

[1] Koenen RR. Lysophosphatidylcholine in Platelet Microvesicles: The Grease for Cardiovascular Disease. Thromb Haemost. 2019; 119:1202–4. 10.1055/s-0039-1693024 31266081

[2] Yadav J, Ismaeel S, Qadri A. Lysophosphatidylcholine Potentiates Antibacterial Activity of Polymyxin B. Antimicrob Agents Chemother. 2020; 64:e01337–20. 10.1128/AAC.01337-20 32988824 PMC7674032

[3] Song MH, Gupta A, Kim HO, Oh K. Lysophosphatidylcholine aggravates contact hypersensitivity by promoting neutrophil infiltration and IL17 expression. BMB Rep. 2021; 54:203–8. 10.5483/BMBRep.2021.54.4.193 33172544 PMC8093940

[4] Liu P, Zhu W, Chen C, Yan B, Zhu L, Chen X, Peng C. The mechanisms of lysophosphatidylcholine in the development of diseases. Life Sci. 2020; 247:117443. 10.1016/j.lfs.2020.117443 32084434

[5] Tao M, Luo J, Gu T, Yu X, Song Z, Jun Y, Gu H, Han K, Huang X, Yu W, Sun S, Zhang Z, Liu L, et al. LPCAT1 reprogramming cholesterol metabolism promotes the progression of esophageal squamous cell carcinoma. Cell Death Dis. 2021; 12:845. 10.1038/s41419-021-04132-6 34518524 PMC8438019

[6] Cotte AK, Aires V, Ghiringhelli F, Delmas D. LPCAT2 controls chemoresistance in colorectal cancer. Mol Cell Oncol. 2018; 5:e1448245. 10.1080/23723556.2018.1448245 30250902 PMC6149788

[7] Ke P, Bao X, Liu C, Zhou B, Huo M, Chen Y, Wang X, Wu D, Ma X, Liu D, Chen S. *LPCAT3* is a potential prognostic biomarker and may be correlated with immune infiltration and ferroptosis in acute myeloid leukemia: a pan-cancer analysis. Transl Cancer Res. 2022; 11:3491–505. 10.21037/tcr-22-985 36388050 PMC9641088

[8] Huttlin EL, Bruckner RJ, Navarrete-Perea J, Cannon JR, Baltier K, Gebreab F, Gygi MP, Thornock A, Zarraga G, Tam S, Szpyt J, Gassaway BM, Panov A, et al. Dual proteome-scale networks reveal cell-specific remodeling of the human interactome. Cell. 2021; 184:3022–40.e28. 10.1016/j.cell.2021.04.011 33961781 PMC8165030

[9] Połońska A, Jasieniecka-Gazarkiewicz K, You L, Hao X, Klińska S, Gong Y, Banaś A. Diatoms and Plants Acyl-CoA:lysophosphatidylcholine Acyltransferases (LPCATs) Exhibit Diverse Substrate Specificity and Biochemical Properties. Int J Mol Sci. 2021; 22:9056. 10.3390/ijms22169056 34445762 PMC8396554

[10] Shindou H, Shimizu T. Acyl-CoA:lysophospholipid acyltransferases. J Biol Chem. 2009; 284:1–5. 10.1074/jbc.R800046200 18718904

[11] Jasieniecka-Gazarkiewicz K, Demski K, Lager I, Stymne S, Banaś A. Possible Role of Different Yeast and Plant Lysophospholipid:Acyl-CoA Acyltransferases (LPLATs) in Acyl Remodelling of Phospholipids. Lipids. 2016; 51:15–23. 10.1007/s11745-015-4102-0 26643989 PMC4700060

[12] Han C, Yu G, Mao Y, Song S, Li L, Zhou L, Wang Z, Liu Y, Li M, Xu B. LPCAT1 enhances castration resistant prostate cancer progression via increased mRNA synthesis and PAF production. PLoS One. 2020; 15:e0240801. 10.1371/journal.pone.0240801 33137125 PMC7605678

[13] Cotte AK, Aires V, Fredon M, Limagne E, Derangère V, Thibaudin M, Humblin E, Scagliarini A, de Barros JP, Hillon P, Ghiringhelli F, Delmas D. Lysophosphatidylcholine acyltransferase 2-mediated lipid droplet production supports colorectal cancer chemoresistance. Nat Commun. 2018; 9:322. 10.1038/s41467-017-02732-5 29358673 PMC5778070

[14] Thomas C, Jalil A, Magnani C, Ishibashi M, Queré R, Bourgeois T, Bergas V, Ménégaut L, Patoli D, Le Guern N, Labbé J, Gautier T, de Barros JP, et al. LPCAT3 deficiency in hematopoietic cells alters cholesterol and phospholipid homeostasis and promotes atherosclerosis. Atherosclerosis. 2018; 275:409–18. 10.1016/j.atherosclerosis.2018.05.023 29866392

[15] Bader JE, Voss K, Rathmell JC. Targeting Metabolism to Improve the Tumor Microenvironment for Cancer Immunotherapy. Mol Cell. 2020; 78:1019–33. 10.1016/j.molcel.2020.05.034 32559423 PMC7339967

[16] Petitprez F, Meylan M, de Reyniès A, Sautès-Fridman C, Fridman WH. The Tumor Microenvironment in the Response to Immune Checkpoint Blockade Therapies. Front Immunol. 2020; 11:784. 10.3389/fimmu.2020.00784 32457745 PMC7221158

[17] Zhao L, Liu Y, Zhang S, Wei L, Cheng H, Wang J, Wang J. Impacts and mechanisms of metabolic reprogramming of tumor microenvironment for immunotherapy in gastric cancer. Cell Death Dis. 2022; 13:378. 10.1038/s41419-022-04821-w 35444235 PMC9021207

[18] Yi K, Zhan Q, Wang Q, Tan Y, Fang C, Wang Y, Zhou J, Yang C, Li Y, Kang C. PTRF/cavin-1 remodels phospholipid metabolism to promote tumor proliferation and suppress immune responses in glioblastoma by stabilizing cPLA2. Neuro Oncol. 2021; 23:387–99. 10.1093/neuonc/noaa255 33140095 PMC7992898

[19] Li L, Wang X, Ding Y, Hui N, Su B, Yang M. LPCAT1 acts as an independent prognostic biomarker correlated with immune infiltration in hepatocellular carcinoma. Eur J Med Res. 2022; 27:216. 10.1186/s40001-022-00854-1 36307879 PMC9617428

[20] McGrail DJ, Pilié PG, Rashid NU, Voorwerk L, Slagter M, Kok M, Jonasch E, Khasraw M, Heimberger AB, Lim B, Ueno NT, Litton JK, Ferrarotto R, et al. High tumor mutation burden fails to predict immune checkpoint blockade response across all cancer types. Ann Oncol. 2021; 32:661–72. 10.1016/j.annonc.2021.02.006 33736924 PMC8053682

[21] McGranahan N, Furness AJ, Rosenthal R, Ramskov S, Lyngaa R, Saini SK, Jamal-Hanjani M, Wilson GA, Birkbak NJ, Hiley CT, Watkins TB, Shafi S, Murugaesu N, et al. Clonal neoantigens elicit T cell immunoreactivity and sensitivity to immune checkpoint blockade. Science. 2016; 351:1463–9. 10.1126/science.aaf1490 26940869 PMC4984254

[22] Matsubara T, Tanaka N, Sato M, Kang DW, Krausz KW, Flanders KC, Ikeda K, Luecke H, Wakefield LM, Gonzalez FJ. TGF-β-SMAD3 signaling mediates hepatic bile acid and phospholipid metabolism following lithocholic acid-induced liver injury. J Lipid Res. 2012; 53:2698–707. 10.1194/jlr.M031773 23034213 PMC3494264

[23] Sun H, Li L, Li W, Yang F, Zhang Z, Liu Z, Du W. p53 transcriptionally regulates SQLE to repress cholesterol synthesis and tumor growth. EMBO Rep. 2021; 22:e52537. 10.15252/embr.202152537 34459531 PMC8490977

[24] Ndiaye H, Liu JY, Hall A, Minogue S, Morgan MY, Waugh MG. Immunohistochemical staining reveals differential expression of ACSL3 and ACSL4 in hepatocellular carcinoma and hepatic gastrointestinal metastases. Biosci Rep. 2020; 40:BSR20200219. 10.1042/BSR20200219 32286604 PMC7198044

[25] Zheng S, Lin J, Pang Z, Zhang H, Wang Y, Ma L, Zhang H, Zhang X, Chen M, Zhang X, Zhao C, Qi J, Cao L, et al. Aberrant Cholesterol Metabolism and Wnt/β-Catenin Signaling Coalesce via Frizzled5 in Supporting Cancer Growth. Adv Sci (Weinh). 2022; 9:e2200750. 10.1002/advs.202200750 35975457 PMC9534957

