# Supplementary material for: Pan-cancer analysis identifies LPCATs family as a prognostic biomarker and validation of LPCAT4/WNT/β-catenin/c-JUN/ACSL3 in hepatocellular carcinoma
Source: Aging (Albany NY). 2023 May 23;15(11):4699–713. doi: 10.18632/aging.204723 (PMC10292872; doi:10.18632/aging.204723)
Supplement: Supplementary Figure 1 [file aging-15-204723-s001.pdf]

SUPPLEMENTARY FIGURE

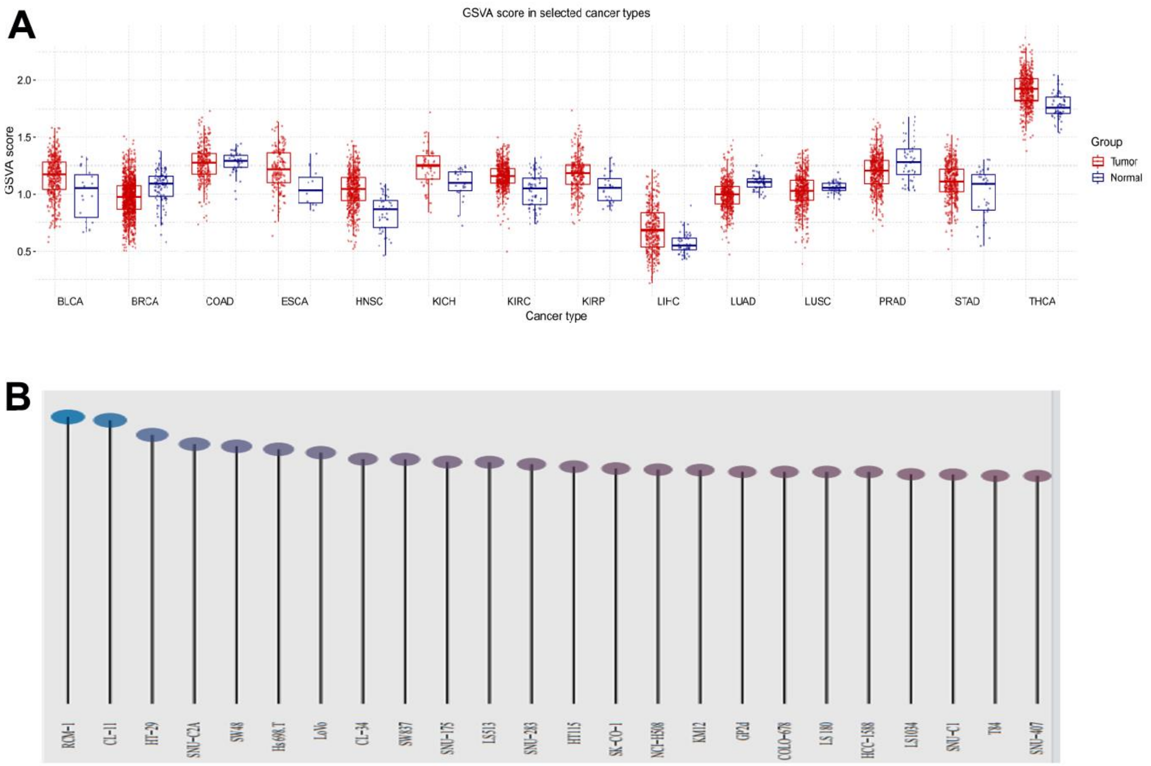

Supplementary Figure 1. (**A**) The LPCATs score in tumors and normal tissues was demonstrated. (**B**) LPCAT4 expression level in LIHC cell lines was demonstrated.
