# Supplementary material for: Pan-cancer analysis identifies LPCATs family as a prognostic biomarker and validation of LPCAT4/WNT/β-catenin/c-JUN/ACSL3 in hepatocellular carcinoma
Source: Aging (Albany NY). 2023 May 23;15(11):4699–713. doi: 10.18632/aging.204723 (PMC10292872; doi:10.18632/aging.204723)
Supplement: Supplementary Table 3 [file aging-15-204723-s004.pdf]

Supplementary Table 3. Primers used for PCR assay.

Gene	Forward primer	Reverse primer
GAPDH	GGAGCGAGATCCCTCCAAAAT	GGCTGTTGTCATACTTCTCATGG
LPCAT4	TCTCGCCTCCAGAGGGTTAAG	AAGAGGACGATAAAGGCCAGA
ACSL3	GCCGAGTGGATGATAGCTGC	ATGGCTGGACCTCCTAGAGTG
c-JUN biding site 1	TGAGGGGAATTACTACTGTG	TTACTGGTGTGAGGGTTGCTTG
c-JUN biding site 2	GCCTGCTCCCCGCCCCTCGG	TCCTGTGTAGCTGGTCTGAC
c-JUN biding site 3	CCGGGCCGCTTGCTACCATTCCA	CCTTGTTTTCTTCCTTGAAAA
